# Physiological, Proteomic Analysis, and Calcium-Related Gene Expression Reveal *Taxus wallichiana* var. *mairei* Adaptability to Acid Rain Stress Under Various Calcium Levels

**DOI:** 10.3389/fpls.2022.845107

**Published:** 2022-03-21

**Authors:** Wen-Jun Hu, Ting-Wu Liu, Chun-Quan Zhu, Qian Wu, Lin Chen, Hong-Ling Lu, Chen-Kai Jiang, Jia Wei, Guo-Xin Shen, Hai-Lei Zheng

**Affiliations:** ^1^Zhejiang Academy of Agricultural Sciences, Hangzhou, China; ^2^School of Life Science, Huaiyin Normal University, Huai’an, China; ^3^State Key Laboratory of Rice Biology, China National Rice Research Institute, Hangzhou, China; ^4^Key Laboratory for Subtropical Wetland Ecosystem Research of Ministry of Education (MOE), College of the Environment and Ecology, Xiamen University, Xiamen, China

**Keywords:** plant proteomics, acid rain, calcium, *Taxus wallichiana var. mairei*, soil, tree species

## Abstract

As one of the serious environmental problems worldwide, acid rain (AR) has always caused continuous damage to the forestry ecosystem. Studies have shown that AR can leach calcium ions from plants and soil. Calcium (Ca) is also a crucial regulator of the plant stress response, whereas there are few reports on how Ca regulates the response of AR-resistant woody plants to AR stress. In this study, by setting different exogenous Ca levels, we study the physiological and molecular mechanism of Ca in regulating the *Taxus wallichiana* var. *mairei* response to AR stress. Our results showed that low Ca level leads to photosynthesis, and antioxidant defense system decreases in *T*. *wallichiana* var. *mairei* leaves; however, these negative effects could be reversed at high Ca level. In addition, proteomic analyses identified 44 differentially expressed proteins in different Ca level treatments of *T*. *wallichiana* var. *mairei* under AR stress. These proteins were classified into seven groups, which include metabolic process, photosynthesis and energy pathway, cell rescue and defense, transcription and translation, protein modification and degradation, signal transduction, etc. Furthermore, the study found that low Ca level leads to an obvious increase of Ca-related gene expression under AR stress in *T*. *wallichiana* var. *mairei* using qRT-PCR analyses and however can be reversed at high Ca level. These findings would enrich and extend the Ca signaling pathways of AR stress in AR-resistant woody plants and are expected to have important theoretical and practical significance in revealing the mechanism of woody plants tolerating AR stress and protecting forestry ecosystem in soil environment under different Ca levels.

## Introduction

As a serious environmental problem, acid rain (AR) affects seriously normal growth and development of forest tree species, which includes balance of leaf nutrient, growth indices, chlorosis, and necrosis in leaves, chlorophyll content, restrict photosynthesis, and the antioxidant enzyme activity (Liu and Diamond, 2005; Larssen et al., 2006; Hu et al., 2014a,^2016^, ^2021^; Huang et al., 2019; Liu M. et al., 2019; Grennfelt et al., 2020; Zhang et al., 2020). Furthermore, AR can interfere with the material metabolism, transcriptional factors, and secondary metabolites genes, further cause metabolic disorders and inhibit the plant growth and development, and even cause plant death in severe cases (Lee et al., 2006; Hu et al., 2016; Debnath and Ahammed, 2020; Debnath et al., 2020, 2021). In addition, recent studies have found that AR severely affects woody plant vegetation growth through leaching away calcium (Ca) element from plant and soil (Likens et al., 1996; Malakoff, 2010; Liu et al., 2011c). Further studies have also revealed that AR can disturb mineral ion absorption, deplete soil base Ca ion from pools, and limit the Ca uptake in woody plant (Liu et al., 2011c; Hu et al., 2014c; Shu et al., 2019). Many studies have demonstrated that Ca plays important roles in plants responding to environmental stresses, and exogenous Ca level regulates cytosolic Ca concentration and enhances antioxidant defense, thus serving as a source of nutrition and structure, regulatory agent to further modulate signaling functions (Reddy and Reddy, 2004; Hepler, 2005; Juice et al., 2006; Zhang et al., 2021). On the other hand, our previous studies also indicated that exogenous Ca level played a key role in the response process of AR stress in AR-sensitive woody plants (Hu et al., 2014c,^2016^). Ma et al. (2021) also found that exogenous Ca enhances antioxidant defense to simulated AR stress in rice. However, the AR-resistant woody plants in response to AR stress at different Ca levels have not been reported in forest tree species.

*Taxus wallichiana* var. *mairei* is a well-known gymnosperm with great ornamental and medicinal value (Gao et al., 2007; Cheng et al., 2021; Xiong et al., 2021). As an AR-resistant tree species, *T. wallichiana* var. *mairei* is distributed over large areas of southern China, where AR is relatively serious (Gao et al., 2007; Hu et al., 2014a). Some physiological and biochemical changes and growth response to simulated AR have been reported in *T. wallichiana* var. *mairei* (Liu K. et al., 2007; Liu et al., 2012). Our previous study also found that *T. wallichiana* var. *mairei* is an AR-tolerant species under normal Ca condition (Hu et al., 2014a). However, the molecular mechanisms of AR resistance remain poorly understood in *T. wallichiana* var. *mairei* under different Ca conditions.

As responses of different Ca levels to AR stress are very complex processes, and further investigations are needed to clarify the molecular mechanism in AR-resistant species. Although our recent study revealed that there are rescue effects of exogenous Ca against AR stress in AR-sensitive species (Hu et al., 2014c,^2016^), the underlying mechanisms remain unclear in AR-tolerant species at various Ca levels. To better understand the extent to which biological and environmental factors shape AR resistance of *T. wallichiana* var. *mairei* at different Ca levels, proteomics and Ca-related gene expression in *T. wallichiana* var. *mairei* response to AR stress at different Ca levels is one of powerful ways to identify the molecular mechanism. A recent study has presented a reference genome of *T. wallichiana* var. *mairei*, which will provide genetic resources and serve as a platform for identification and decoding of the AR resistance pathway in various Ca conditions in the future (Cheng et al., 2021; Xiong et al., 2021). In this study, we conduct proteomic study to clarify the molecular mechanisms of various Ca levels in *T. wallichiana* var. *mairei* under AR treatment. The objective of this study was to characterize the AR-responsive proteins in *T. wallichiana* var. *mairei* at different Ca levels, combined with the physiological and gene expression data, and further to establish the molecular metabolism in Ca-mediated AR resistance in AR-resistant woody plants.

## Materials and Methods

### Soil Pretreatment

In our experiment, the substrate soil was lateritic soil, and the soil samples were collected from in southern forest areas of China where AR is much too harmful. Ca content in the soil was leached for 6 months of simulated AR, and the specific soil leaching process is referred to Liu J. X. et al. (2007) and Liu et al. (2011c). According to Hu et al. (2014c), Ca content was analyzed using ICP-MS (PerkinElmer Inc., Elan DRC-e, Waltham, MA, United States). Soil nutrients were recovered by a Hoagland nutrient solution, which contains one of the three Ca concentrations (20.0, 2.0, or 0.1 mmol L^–1^), respectively (Liu et al., 2011c). The final soil exchangeable Ca level was high Ca level (107.08 mmol kg^–1^), medium Ca level (19.65 mmol kg^–1^), and low Ca level (1.85 mmol kg^–1^).

### Plant Materials and Experimental Procedure

The 6-month-old and size-identical *T. wallichiana* var. *mairei* seedlings (the aerial part length was 6.3 ± 0.5 cm) were transplanted into plastic pots. The seedlings were grown in a greenhouse with a light–dark regime of 16/8 h, temperature of 27/21°C (day/night), relative humidity of 60–70%, and photosynthetically active radiation of 210 μmol m^–2^ s^–1^. After 2 weeks of recovery, the *T. wallichiana* var. *mairei* seedlings were sprayed once each day with simulated AR solution (pH 3.0) with medium Ca level as control group and low or high Ca level for treatment group as described by the previous study (Hu et al., 2014c). After 2-month AR treatment, the fresh leaves of *T. wallichiana* var. *mairei* seedlings were collected for further experiments, which include physiological and proteomic research and qRT-PCR analysis.

### Measurements of Physiological and Growth Indexes

Chlorophyll in *T. wallichiana* var. *mairei* leaves was extracted using ice-cold 80% v/v acetone according to the study by Hu et al. (2014c). Net photosynthetic rate (*P*n) was performed with a portable photosynthesis system (Li-6400, Li-Cor, Lincoln, NE, United States) as described by Chen et al. (2013). At least eight saplings were randomly selected from the control group or the treatment group for *P*n measurements. For Ca element analysis, T. wallichiana var. mairei leaf tissue was dried at 80°C for 72 h. Ca concentration in the leaves was measured using ICP-MS (PerkinElmer Inc., Elan DRC-e, Waltham, MA, United States), following the study by Hu et al. (2014c).

### Assay of Lipid Peroxidation, Reactive Oxygen Species Production, and Antioxidant Enzyme Activity

Soluble protein, proline, H_2_O_2_, and O2^•–^ content were measured as described by Chen et al. (2013). According to Liu et al. (2011a), lipid peroxidation in leaves was measured by estimating the content of malondialdehyde (MDA) using thiobarbituric acid (TBA) reaction. Superoxide dismutase (SOD) activity, ascorbic peroxidase (APX) activity, peroxidase (POD) activity, and catalase (CAT) activity were measured following the methods of Hu et al. (2014b).

### Protein Extraction, Two-Dimensional Electrophoresis, and Data Analysis

Protein in *T. wallichiana* var. *mairei* leaves was extracted using the method of phenol extraction according to the study of Hu et al. (2014a). Three independent biological repetitions were performed for each treatment. According to the manufacturer’s instructions of GE Healthcare Amersham Bioscience, protein concentration of the lysates was measured using a 2-D Quant Kit.

Two-dimensional electrophoresis (2-DE) was conducted according to the study of Hu et al. (2014a). Ettan IPGphor isoelectric focusing system (GE Healthcare Amersham Bioscience, Little Chalfont, United Kingdom) was used for isoelectric focusing (IEF). After IEF, gel strips were equilibrated as described by Hu et al. (2014c). For the second-dimension electrophoresis, it was conducted using a protein apparatus (Bio-Rad), the proteins were separated on 12.5% SDS polyacrylamide gels according to the manufacturer’s instructions (Hu et al., 2014c). The 2-DE gels were stained using Coomassie Brilliant Blue R-250, and an image scanner (Uniscan M3600, China) was used for gel images at 600 dots per inch resolution. The 2-D gel images were analyzed using PDQuest software (Version 8.01, Bio-Rad, Hercules, CA, United States). The intensity of protein spots changed more than twofold and passed the Student’s *t*-test (*p* < 0.05), which were considered for mass spectrometry analysis.

### Protein Identification and Protein Classification

Protein digestion and protein identification in *T. wallichiana* var. *mairei* was performed according to the study of Hu et al. (2014a). The identified tryptic peptide masses in *T. wallichiana* var. *mairei* were searched against the National Center for Biotechnology Information non-redundant (NCBInr) database, and the taxonomy of green plants was selected using the MASCOT interface (Version 2.5; Matrix Science, London, United Kingdom). The following parameters were used for database search: no molecular weight restriction, permitting one missed cleavage, fixed modification of cysteine by carbamidomethylation, oxidation (Met) as a variable modification, the peptide tolerance of 100 ppm, and fragment ion mass tolerance of ±0.3 Da. At least three peptides were matched for protein identification, and the protein scores of MOWSE threshold were set greater than 73 with the NCBInr database (*p* < 0.05). As described by Hu et al. (2014a), the functions and subcellular localization of identified proteins were searched for the NCBI protein database,^1^ UniProt,^2^ and published literature.

### Hierarchical Cluster Analysis

Hierarchical clustering was performed on density value of differentially expressed proteins according to the study of Hu et al. (2014c). Input value was calculated by dividing volume percentage of each protein spot at the high Ca-AR level and low Ca-AR level by the corresponding protein spot at the medium Ca-AR level. Complete linkage algorithm was enabled, and the results of hierarchical cluster were plotted using TreeView software version 1.1.3.

### RNA Extraction and qRT-PCR Analysis

Total RNA from *T. wallichiana* var. *mairei* leaves (0.1 g) was extracted using RNA purification reagent (Invitrogen Inc., CA, United States) in liquid nitrogen according to the study of Hu et al. (2014c). The M-MLV reverse transcriptase (TaKaRa, Dalian, China) was used for the synthesis of the first-strand cDNAs as described by the previous study (Hu et al., 2014c). Gene primers were designed for cloning the fragments of Ca-related genes in *T. wallichiana* var. *mairei* (Supplementary Table 1). The Ca-related gene abundance was analyzed using the Rotor-gene-6000 real-time PCR system (Corbett Research, Mortlake, NSW, Australia) as described by Hu et al. (2014c) with minor modifications. The following temperature program was used for qRT-PCR analysis: 94°C for 10 min, followed 94°C for 30 s by 40 cycles, primer annealing at 52–56°C for 30 s (Supplementary Table 2), and extension at 72°C for 20 s. The glyceraldehyde-3-phosphate dehydrogenase gene (*GAPDH*) in *T. wallichiana* var. *mairei* was used as the internal control for each sample. Three independent biological replicates were performed for each sample.

### Statistical Analysis

All data were presented as the mean ± SE of three replicated samples. The statistical significance was analyzed using a univariate analysis of variance (one-way ANOVA; SPSS, version 22.0, Inc., Chicago, IL, United States). Statistical significance was considered at *p* < 0.05.

## Results

### Effects of Acid Rain Stress on Physiological Parameters of *Taxus wallichiana* var. *mairei* at Different Calcium Levels

**FIGURE 1:**
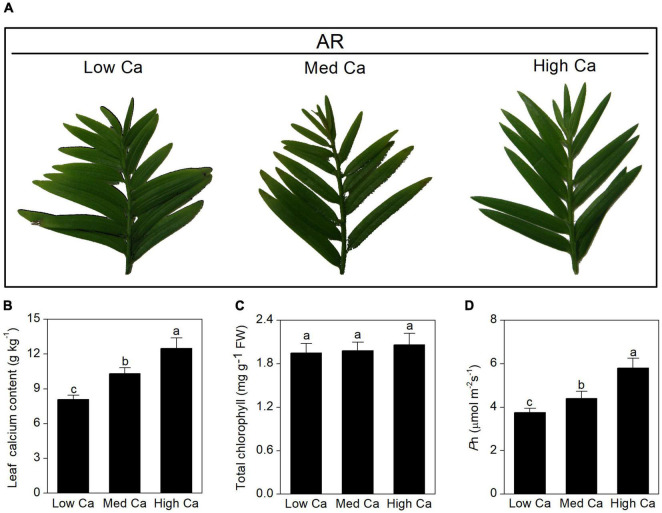
Effects of different Ca treatments on morphological and physiological parameters of *T. wallichiana* var. *mairei* leaves under AR stress. **(A)** Phenotype on plant leaves. **(B)** Leaf Ca content. **(C)** Chlorophyll content. **(D)** Net photosynthetic rate (*P*n). Different letters above columns indicate significant difference at *p* < 0.05.

To study the responses of woody plant to AR stress at different Ca levels, *T. wallichiana* var. *mairei* was treated with simulated AR (pH 3.0) for 2 months. Physiological changes are shown in Figure 1, and Ca content of the leaf, chlorophyll content, and photosynthetic activity (*P*n) were also tested and analyzed. After 2-month AR treatment, Ca content and *P*n were significantly decreased in *T. wallichiana* var. *mairei* leaves at low Ca level; however, high Ca treatment can reverse the decline of the physiological indicators.

### Effects of Acid Rain Stress on Antioxidant System Response in *Taxus wallichiana* var. *mairei* at Different Calcium Levels

**FIGURE 2:**
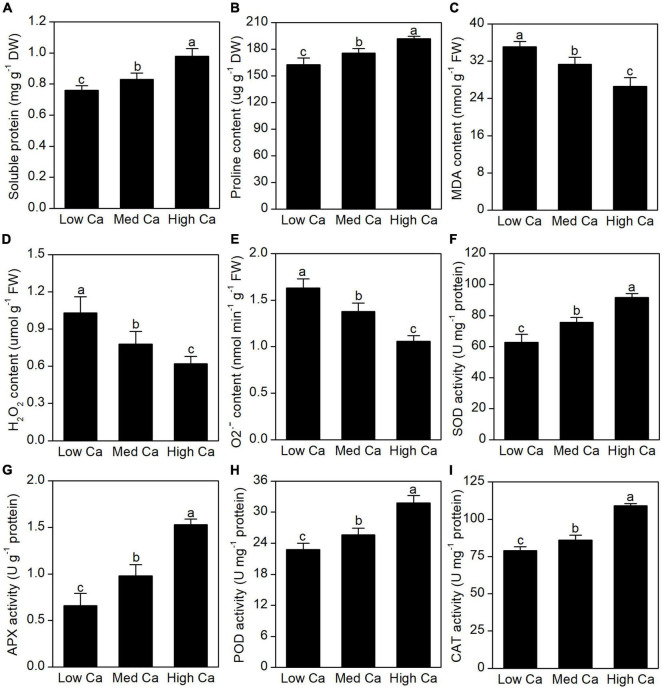
Effects of different Ca treatments on lipid peroxidation, ROS production, and antioxidant enzyme activity under simulated AR stress in *T. wallichiana* var. *mairei* leaves. The soluble protein content **(A)**, proline content **(B)**, MDA content **(C)**, H_2_O_2_ content **(D)**, O2^•–^ content **(E)**, SOD activity **(F)**, APX activity **(G)**, POD activity **(H)**, and CAT activity **(I)**. Columns labeled by different letters indicate significant differences at *p* < 0.05.

As shown in Figures 2A,B, there was an obvious increase in soluble protein content and proline content in AR-treated *T. wallichiana* var. *mairei* at high Ca level. We found that the levels of MDA, H_2_O_2_, and O2^•–^ were significantly stimulated by low Ca treatment (Figures 2C–E). It found that SOD, APX, POD, and CAT showed a significant increase in *T. wallichiana* var. *mairei* leaves at high Ca level (Figures 2F–I).

### Protein Profile and Functional Classification in *Taxus wallichiana* var. *mairei* Leaves Response to Acid Rain Stress at Different Calcium Levels

To further explore the proteome changes in *P*inus *massoniana* and *T. wallichiana* var. *mairei* leaves under AR treatment, 2-DE was performed in this study.

**FIGURE 3:**
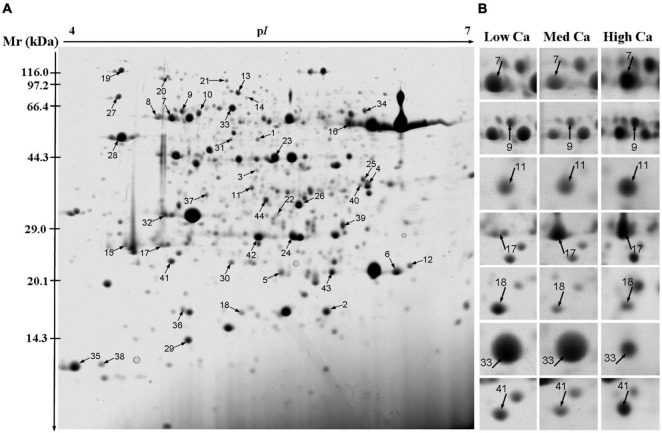
Two-dimensional electrophoresis analysis of proteins extracted from the leaves of *T. wallichiana* var. *mairei* treated at different Ca levels under AR stress. The numbers are assigned to the protein spots correspond to those listed in Table 1. **(A)** Representative CBB R250-stained 2D gel of total proteins. Arrows indicate 44 spots showing at least twofold changes (*p* < 0.05) analyzed by MALDI-TOF/TOF MS. **(B)** The enlarged window represents some differentially expressed protein spots after AR treatment at different Ca levels.

**FIGURE 4:**
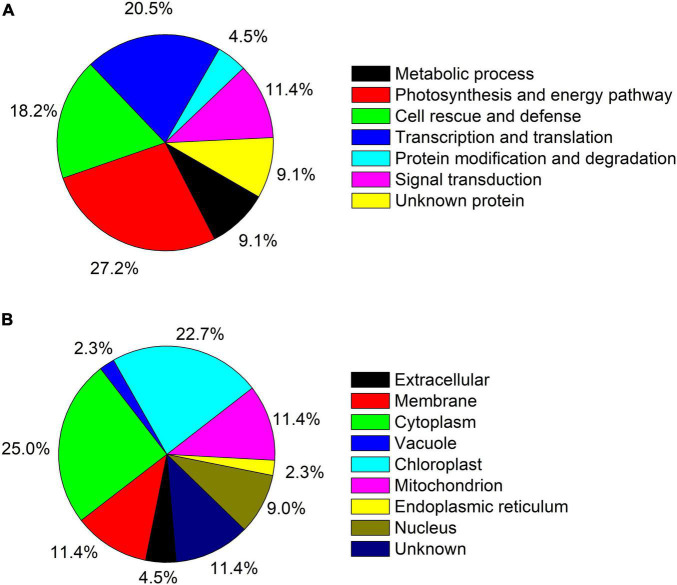
**(A)** Functional category distribution of the identified proteins in AR-treated *T. wallichiana* var. *mairei* at low, medium, and high Ca levels. **(B)** Protein subcellular locations of all 44 identified and quantified proteins in AR-treated *T. wallichiana* var. *mairei* at low, medium, and high Ca levels.

A total of 44 protein spot in *T. wallichiana* var. *mairei* leaves response to AR stress with different Ca treatments (Figure 3A and Table 1). Close-up views of representative different protein spots are shown in Figure 3B. To analyze which biological processes the identified differential proteins are involved in, the identified 44 proteins were analyzed in appropriate functional pathways (Figure 4). The differentially expressed proteins were divided into seven groups based on their biological functions. The largest protein group was sorted to photosynthesis and energy pathway (27.2%), followed by transcription and translation (20.5%), cell rescue and defense (18.2%), signal transduction (11.4%), metabolic process (9.1%), and protein modification and degradation (4.5%) (Figure 4A). As shown in Figure 4B, the subcellular localization analysis found that 44 differentially expressed proteins were located in the cytoplasm (25.0%), chloroplast (22.7%), mitochondrion (11.4%), membrane (11.4%), nucleus (9.0%), extracellular (4.5%), endoplasmic reticulum (2.3%), and vacuole (2.3%).

**TABLE 1:** Differentially expressed proteins of *Taxus wallichiana* var. *mairei* in response to AR stress under different calcium levels.

Spot^a^	NCBI accession^b^	Protein identity^c^	Thero. kDa/p*I*^d^	Exper. kDa/p*I*^e^	Pep. Count^f^	Score^g^
**Metabolic process**						
1	gi| 2500930	Cell wall beta-fructosidase 1	62.90/7.07	46.39/5.49	6	94
2	gi| 158705664	UDP-glucose pyrophosphorylase	51.97/5.34	11.69/5.91	6	90
4	gi| 223635315	S-adenosylmethionine synthase	43.61/5.55	25.36/6.17	7	107
5	gi| 77556698	Phytoene synthase	44.99/8.64	15.91/5.67	5	81
**Photosynthesis and energy pathway**						
7	gi| 138277483	ATP synthase beta subunit	51.67/5.11	62.61/4.99	18	164
8	gi| 138277483	ATP synthase beta subunit	51.67/5.11	63.61/4.90	18	170
9	gi| 4388533	F1-ATP synthase beta subunit	49.22/5.25	67.99/5.04	16	174
10	gi| 226493589	ATP synthase beta chain	59.06/5.90	65.39/5.14	12	125
11	gi| 7592732	Plasma membrane H^+^-ATPase	22.04/8.92	31.30/5.47	6	102
12	gi| 372486191	NADH-plastoquinone oxidoreductase subunit 7	46.04/5.78	25.36/6.53	6	81
13	gi| 311893429	ATP-dependent zinc metalloprotease ThFtsH8	73.83/6.33	83.00/5.37	10	108
14	gi| 356517518	ATP-dependent zinc metalloprotease FTSH	74.58/5.93	77.61/5.42	14	134
15	gi| 3293555	Chlorophyll *a*/*b* binding protein	28.35/5.47	20.90/4.74	6	93
16	gi| 91177512	Ribulose-1,5-bisphosphate carboxylase/oxygenase large subunit	51.99/6.23	25.36/6.04	16	150
17	gi| 224114357	Light-harvesting complex II protein Lhcb1	28.09/5.29	20.55/4.94	7	96
18	gi| 3913651	Ferredoxin-NADP reductase	40.71/8.37	11.75/5.40	6	88
**Cell rescue and defense**						
19	gi| 255570990	Heat shock protein	75.43/5.35	120.00/4.69	8	92
20	gi| 392465167	Heat shock protein 70	71.46/5.14	107.67/4.97	17	178
21	gi| 357493781	Thioredoxin-related protein	32.06/7.74	98.05/5.30	5	91
22	gi| 110808557	Trypsin inhibitor AeTI	2.24/4.55	25.13/5.63	3	85
23	gi| 153865891	Alcohol dehydrogenase 1	21.73/6.23	39.43/5.60	5	96
24	gi| 327342604	Glutathione S-transferase	25.52/6.34	21.12/5.71	6	96
25	gi| 335346406	Abscisic acid 8-hydroxylase	53.18/8.77	25.36/6.17	7	88
26	gi| 302815799	2-Oxoglutarate-iron(II)-dependent oxygenase	40.96/5.76	26.72/5.75	6	90
**Transcription and translation**						
27	gi| 14579025	Maturase K	60.80/9.56	85.64/4.67	10	99
28	gi| 56744289	Putative transposon MuDR mudrA-like protein	85.62/8.03	49.90/4.69	9	106
29	gi| 67968326	Ribosomal protein L14	13.07/10.40	10.00/5.10	5	90
30	gi| 62733886	Retrotransposon protein	64.07/7.58	17.28/5.35	7	87
31	gi| 379054892	Initiation factor 4A-3-like protein	43.02/6.30	46.73/5.33	10	123
32	gi| 359494561	Transcription factor JUNGBRUNNEN 1	32.65/6.67	26.35/4.95	6	89
33	gi| 159480324	Mitochondrial transcription termination factor	25.87/9.46	68.05/5.34	6	106
34	gi| 77551510	Transposon protein	81.78/9.17	25.36/6.13	10	112
35	gi| 334183835	Small subunit ribosomal protein S1	56.63/5.06	8.46/4.47	8	96
**Protein modification and degradation**						
6	gi| 385178691	Probable aspartyl aminopeptidase	54.33/6.36	25.36/6.37	6	88
36	gi| 357469355	F-box/kelch-repeat protein	48.50/6.29	11.99/5.08	7	100
**Signal transduction**						
3	gi| 269980525	IAA-amino acid hydrolase	47.90/5.95	35.80/5.48	6	96
37	gi| 195627742	Membrane steroid-binding protein 1	10.99/5.35	30.34/5.20	4	84
38	gi| 356516069	2A phosphatase-associated protein of 46 kDa	45.15/5.39	8.59/4.58	11	111
39	gi| 307939386	Lectin	9.86/9.23	25.36/6.02	4	81
40	gi| 356573251	Calcium-binding protein KIC-like	14.00/4.18	25.36/6.13	7	112
**Unknown protein**						
41	gi| 116782579	Unknown	15.71/6.30	17.74/5.01	4	74
42	gi| 356566253	Uncharacterized protein LOC100799858	38.98/6.01	20.96/5.52	6	98
43	gi| 296087931	Unnamed protein product	79.30/7.32	15.63/5.96	11	106
44	gi| 20198271	Hypothetical protein	13.97/7.88	28.14/5.54	5	91

*^a^Spot is the unique differentially expressed protein spot number.*

*^b^Database accession numbers according to NCBInr.*

*^c^The name of the proteins identified by MALDI-TOF/TOF MS.*

*^d^Theoretical mass (kDa) and pI of identified proteins.*

*^e^Experimental mass (kDa) and pI of identified proteins.*

*^f^Number of the matched peptides.*

*^g^The Mascot searched score against the database NCBInr.*

**FIGURE 5:**
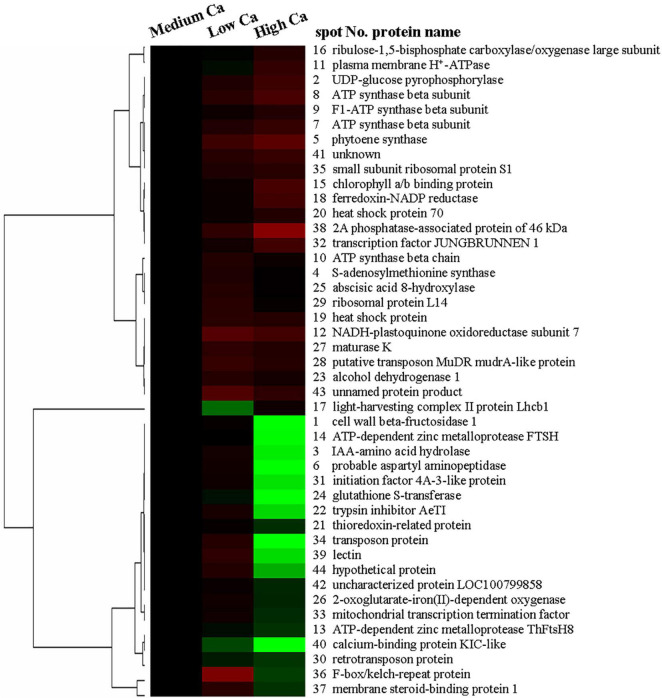
Hierarchical clustering analyses of the 44 AR-responsive proteins expression profiles at different Ca levels in *T. wallichiana* var. *mairei.*

In addition, we used a hierarchical cluster analysis to find the regular pattern of different protein expression changes during AR stress in *T. wallichiana* var. *mairei* leaves with different Ca treatments. As shown in Figure 5, the differentially expressed proteins are mainly distributed into two branches in *T. wallichiana* var. *mairei*. We found that the expression abundance of cell defense-related proteins (spots 21, 22, and 24) showed a downward trend with the increase of Ca concentration under AR stress. Signal transduction-related proteins (spots 3, 37, and 39) showed a significant upregulation trend under low Ca-AR treatment. The expression trend of these proteins was reversed to downregulated with the increase in Ca concentration. More interestingly, we found that a protein (spot 17, light-harvesting complex II protein Lhcb1) related to photosynthesis process was significantly downregulated in low Ca condition, but turned to upregulated in high Ca condition, which indicates that high Ca treatment helps to improve *T. wallichiana* var. *mairei* leaves photosynthesis, which is one of the ways to enhance plant resistance to AR stress. The specific information of the differential proteins is listed in Table 1.

### Protein Abundance Analysis by Western Blot

**FIGURE 6:**
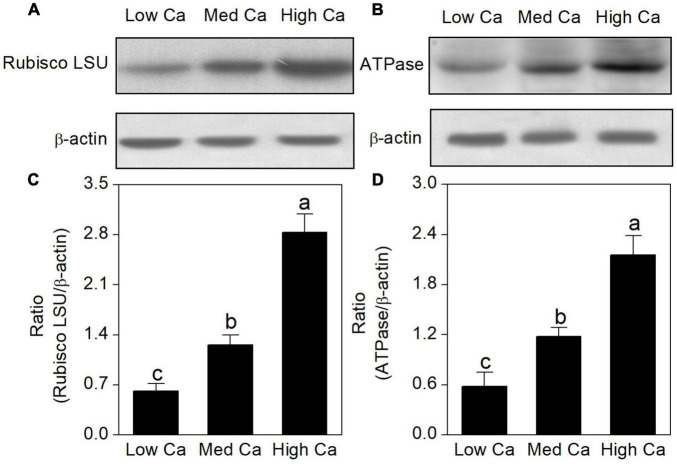
Effects of different Ca levels on rubulose-1, 5-bisphoshate carboxylase–oxygenase large subunit (Rubisco LSU) **(A)**, ATP synthase (ATPase) **(B)** in *T. wallichiana* var. *mairei* under AR stress using western blot. Relative expression levels of Rubisco LSU **(C)** and ATPase **(D)** were analyzed with the Quantity One software. β-Actin was used as the internal control. Bars with different letters are significantly different from each other (*p* < 0.05).

The proteomics results revealed that the abundance of ribulose-1, 5-bisphosphate carboxylase–oxygenase large subunit (spot 16), and ATP synthase (spots 7, 8, and 10) was increased in high Ca-AR-treated *T. wallichiana* var. *mairei* (Table 1). As shown in Figure 6, protein abundance levels of rubulose-1, 5-bisphoshate carboxylase–oxygenase large subunit (Rubisco LSU), and ATP synthase (ATPase) were significantly increased with high Ca treatment.

### Calcium-Related Gene Expression Analysis

**FIGURE 7:**
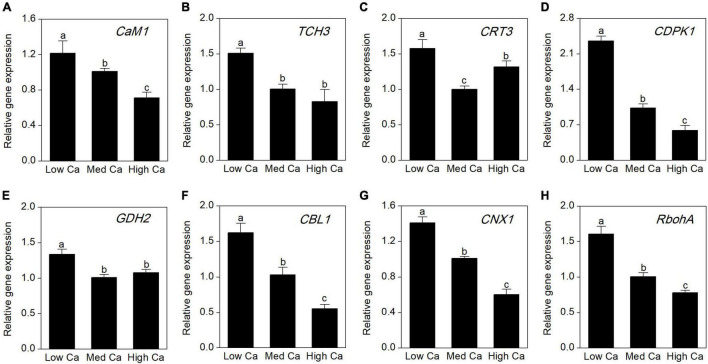
Expression analysis by qRT-PCR of eight Ca-related genes in *T. wallichiana* var. *mairei* treated at different Ca levels under AR stress. **(A)** Calmodulin 1 (*CaM1*), **(B)** touch 3 gene (*TCH3*), **(C)** calreticulin 3 gene (*CRT3*), **(D)** calcium-dependent protein kinase 1 (*CDPK1*), **(E)** glutamate dehydrogenase 2 gene (*GDH2*), **(F)** calcineurin B-like calcium sensor protein 1 gene (*CBL1*), **(G)** calnexin 1 gene (*CNX1*), **(H)** respiratory burst oxidase homolog A gene (*RbohA*). Bars represent the mean value ± SE (*n* = 3). Bars with different letters are significantly different from each other (*p* < 0.05).

To evaluate the expression abundances of the Ca-related genes at different Ca levels in AR-treated *T. wallichiana* var. *mairei*, we further analyze calmodulin gene (*CaM1*), touch 3 gene (*TCH3*), calreticulin 3 gene (*CRT3*), CDPK-related kinase gene (*CDPK1*), glutamate dehydrogenase 2 gene (*GDH2*), calcineurin B-like calcium sensor protein 1 gene (*CBL1*), calnexin 1 gene (*CNX1*), and respiratory burst oxidase homolog A gene (*RbohA*). As shown in Figure 7, under AR stress, the expression abundance of most Ca-related genes increased obviously at low Ca level; however, this expression trend can be reversed at high Ca level.

## Discussion

### Metabolic Process, Protein Modification, and Degradation-Related Proteins

The environmental stresses severely affect the material metabolism of plants (Liu et al., 2011a; Chen et al., 2014). UDP-glucose pyrophosphorylase is the precursor of carbohydrate formation (Chen et al., 2019). Our results showed that Ca application reduced AR-induced increases in protein abundance of UDP-glucose pyrophosphorylase (spot 2) in *T. wallichiana* var. *mairei* (Table 1). Under AR stress, our previous study indicates that high Ca level enhances the carbohydrate metabolism in *L*iquidambar *formosana* (Hu et al., 2016). It has been showed that woody plants against environmental stresses with a complex manner, which results in similar protein expressions (Hu et al., 2014c; Liu Y. et al., 2019). In this study, our finding is consistent with the results from the study of how exogenous Ca affects other woody plants to AR stress. These results showed that Ca application helps in alleviating the adverse effects of AR stress by accelerating the carbohydrate metabolism. On the other hand, Chen et al. (2019) found that UDP-glucose pyrophosphorylase plays an important role in cell wall metabolism and the synthesis of cellulase and hemicellulose. Carbohydrates act as a cytoskeleton and compose the cell wall as the first physical barrier defending against all types of AR stress (Liu et al., 2011b). We speculate that the protecting role of Ca can be explained by the upregulated carbohydrate metabolism-related protein under AR stress. S-adenosylmethionine synthase catalyzes the formation of S-adenosylmethionine from methionine and ATP, which is the critical enzymes in ethylene biosynthetic process (Peleman et al., 1989; Wang et al., 2002). In this study, protein abundances of S-adenosylmethionine synthase (spot 4) increased after low Ca treatment (Figure 5), which indicates that Ca-mediated ethylene biosynthetic process might make effect in AR tolerance in *T. wallichiana* var. *mairei*. Additionally, the abundance of S-adenosylmethionine synthase increases in high Ca-AR-treated *L. formosana* (Hu et al., 2016). These findings indicate that exogenous Ca plays different roles in AR-resistant tree species (*T. wallichiana* var. *mairei*) and AR-sensitive tree species (*L. formosana* and *P. massoniana*) in response to AR stress. Further study is needed to clarify the different metabolic processes at various Ca levels in AR-tolerance woody plants.

Aspartyl aminopeptidase is likely to play an important role in intracellular protein proteolysis and peptide metabolism (Park et al., 2017). In addition, F-box/kelch-repeat protein is involved in the pathway of protein ubiquitination, which is a part of protein modification. F-box/kelch-repeat protein may mediate the ubiquitination and subsequent proteasomal degradation of target proteins (González-Carranza et al., 2007). In this study, our proteomic analysis has detected increased abundance of probable aspartyl aminopeptidase (spot 6) and F-box/kelch-repeat protein (spot 36) at low Ca level in *T. wallichiana* var. *mairei* response to AR stress; however, this expression trend can be reversed at high Ca level (Table 1). It should be noted that the previous proteome analysis reveals the significantly elevated abundance of F-box family protein in AR-treated woody plant, which suggests that AR stress affects the biosynthesis and refolding of proteins and exacerbated protein degradation (Chen et al., 2014; Hu et al., 2014a). The findings provide informative clues regarding the protein modification and degradation mechanisms for the negative regulation of exogenous Ca level during *T. wallichiana* var. *mairei* response to AR stress. It is reasonable to speculate that AR stress leads to more serious protein degradation in low Ca condition, and high expression of degraded proteins system needs to be activated to maintain the stability of the protein metabolism in *T. wallichiana* var. *mairei*; high Ca condition has effective protection mechanism, low expression, or without degradation of protein fragments damaged by AR stress. AR stress can damage the homeostasis of protein metabolism between biosynthesis and degradation (Hu et al., 2014a). Most of these proteins play important roles in linking change of Ca concentration and the subsequent metabolic adaptation to AR stress in woody plants (Hu et al., 2014c,^2016^), but the specific details of the mechanism need to be further studied in the future.

### Photosynthesis and Energy Pathway-Related Proteins

Photosynthesis is a key metabolic process of woody plants, which is sensitive to AR stress (Liu et al., 2011a; Chen et al., 2013). In addition, our previous studies found that photosynthesis is greatly affected by simulated AR stress under low Ca level (Hu et al., 2014c,^2016^). In this study, physiological analysis found that the photosynthesis is inhibited with low Ca-AR treatment, but significantly increased with high Ca-AR treatment in *T. wallichiana* var. *mairei* leaves (Figure 1C). Consistent with the results of physiological data, our proteomic data analysis showed that high Ca level can remarkably improve photosynthetic capacity of *T. wallichiana* var. *mairei* with AR treatment. For instance, we found that chlorophyll *a*/*b*-binding protein (spot 15), ribulose-1,5-bisphosphate carboxylase–oxygenase large subunit (spot 16), light-harvesting complex II protein Lhcb1 (spot 17), and ferredoxin-NADP reductase (spot 18) were decreased in protein expression abundance with low Ca-AR treatment and increased with high Ca-AR treatment in *T. wallichiana* var. *mairei* (Table 1). These results indicated that high Ca level could restore the impaired photosynthetic function in AR-treated *T. wallichiana* var. *mairei* seedlings.

The protective role of exogenous Ca may lie in the increased upregulation of photosynthesis and energy pathway, which enhances AR tolerance in *T. wallichiana* var. *mairei*. This inference is supported by our previous studies, in which we demonstrated that exogenous Ca supply improved photosynthesis in woody plants against AR stress (Hu et al., 2014c,^2016^).

On the other hand, sufficient ATP is necessary for *T. wallichiana* var. *mairei* responding to AR stress in plants. ATP synthase and ATPase are the key enzymes in energy production and conversion (Liu et al., 2011b; Hu et al., 2014a). Previous evidence indicates that enough ATP is necessary for plants in response to environmental stress (Jiang et al., 2007). Our results found that a large number of enzymes were involved in this process that include ATP synthase beta subunit (spots 7, 8), F1-ATP synthase beta subunit (spot 9), and ATP synthase beta chain (spot 10). Our previous studies found that AR-sensitive tree species (*L. formosana* and *P. massoniana*) have the increased abundance of energy production-related proteins with high Ca-AR treatment (Hu et al., 2014c,^2016^). In this study, we also find a similar pattern in *T. wallichiana* var. *mairei*. Additionally, western blot analysis showed that relative abundance levels of the Rubisco LSU and ATPase were increased with high Ca treatment (Figure 6), and western blot result is consistent with the selected protein of our proteomic data at various Ca levels in AR-treated *T. wallichiana* var. *mairei*. Besides, as a membrane-bound protein located in thylakoids, ATP-dependent zinc metalloprotease-related proteins involve in the removal of a damaged D1 protein from PSII in plants (Hu et al., 2014a). Expression abundance of ATP-dependent zinc metalloprotease ThFtsH8 (spot 13) and ATP-dependent zinc metalloprotease FTSH (spot 14) was downregulated in high Ca-AR-treated *T. wallichiana* var. *mairei*. This change meant that less damaged protein is produced in high Ca condition, by which we furthermore find that the recovered photosynthesis decline caused by AR stress. Moreover, H^+^-ATPase plays an important role in the maintenance of ion homeostasis and nutrient uptake in plants (Liu et al., 2011b; Chen et al., 2019). Our study also showed that plasma membrane H^+^-ATPase (spot 11) was upregulated by high Ca level with AR treatment. Based on these results, high Ca condition may stimulate the resistance of *T. wallichiana* var. *mairei* to AR stress by modulating special energy-related pathway. These findings indicate that with high Ca-AR treatment, the mechanisms of energy pathway in AR-resistant tree species (*T. wallichiana* var. *mairei*) are different from AR-sensitive tree species (*L. formosana* and *P. massoniana*). Further investigations are needed to clarify the different energy pathways in AR-tolerance woody plants.

### Cell Rescue and Defense-Related Proteins

Acid rain stress has negative effects on plant growth and development, and also the internal functional components. Low Ca-AR treatment increased the content of MDA and reactive oxygen radicals [reactive oxygen species (ROS), e.g., H_2_O_2_ and O2^•–^], which suggests the ROS enrichment in *T. wallichiana* var. *mairei*; however, high Ca supplementation can reverse these effects (Figure 2). In addition, we observed that soluble protein content, proline content, SOD, APX, POD, and CAT activity showed a significant increase in *T. wallichiana* var. *mairei* leaves with high Ca treatment, which implies that antioxidant defense system was provoked by high Ca supplementation in AR-treated *T. wallichiana* var. *mairei*. These changes of physiological and biochemical findings indicated the protective role in exogenous Ca being against AR stress in *T. wallichiana* var. *mairei*.

To further reveal the different strategies to cope with AR stress at various Ca levels, many cell rescue and defense-related proteins were also identified by proteomic analysis in this study. As stress response proteins and heat shock proteins can be induced by many abiotic stresses (Wang et al., 2004, 2013), our study showed that exogenous Ca addition enhanced the abundance of heat shock protein (spot 19) and heat shock protein 70 (spot 20). Additionally, heat shock proteins are also considered as an important part in stabilizing protein folding and play important roles in the cellular stress response (Chen et al., 2019). The increased abundance of the heat shock-related proteins in high Ca-AR treatment set indicated the multidimensional role of external Ca supply in the alleviation of AR stress in *T. wallichiana* var. *mairei*.

In this study, decreased expressions of thioredoxin-related protein (spot 21) and glutathione S-transferase (spot 24) were also observed at high Ca levels. It was reported that thioredoxin can reduce intramolecular disulfide bridges of target proteins, maintain suppression of apoptosis, and supply reducing equivalents to the antioxidant systems (Tada et al., 2008; Liu et al., 2011b). Under both low and high Ca levels, elevated abundance of thioredoxin superfamily protein was described in *L. formosana* seedlings in response to AR stress (Hu et al., 2016). In addition, glutathione S-transferases are associated with stress tolerance, which can catalyze the addition of glutathione to electrophilic compounds and resist various cellular damages (Frova, 2003; Aloui et al., 2009). Previous studies reported the enhanced expressions of glutathione S-transferase in *Arabidopsis* response to AR stress and low Ca-AR-treated *P. massoniana* seedlings (Hu et al., 2014c,^2016^). As an antioxidative protein, glutathione S-transferase can be strongly induced by biotic and abiotic stresses (Dixon et al., 2002). In this study, downregulation of these proteins indicates that high Ca level has the potential to protect *T. wallichiana* var. *mairei* from oxidative stress caused by AR stress, which suggests that AR-resistant tree species has developed different defense strategies against AR stress under high Ca condition.

### Transcription and Translation-Related Proteins

Transcription and translation regulate the expression level of stress responsive genes, which are important for plants response to environmental stresses (Jiang et al., 2007; Chen et al., 2014; Hu et al., 2014c). In this study, we have successfully identified nine proteins related to transcription and translation. In plants, maturase K catalyzes intron RNA binding and affects gene expression at the transcriptional level (Ji et al., 2009). The expression level of maturase K is complex in plants response to abiotic stress, which depends on plant species and the type of environmental stresses (Pandey et al., 2008; Chen et al., 2014). In this study, the abundance of maturase K (spot 27) increases in both low Ca-AR and high Ca-AR-treated *T. wallichiana* var. *mairei*, which suggests that expression changes in maturase K may play a part in linking change of Ca level under AR stress. Moreover, Chen et al. (2019) showed that a transcription factor displayed an elevated expression pattern in Arabidopsis of exogenous Ca-alleviated Al toxicity. We also found that exogenous high Ca enhances the abundance of transcription factor JUNGBRUNNEN 1 (spot 32) in *T. wallichiana* var. *mairei.* In addition, Hu et al. (2016) found that an increase in ribosomal protein S1 was detected in high Ca-AR-treated woody plant. Consistent with previous research, protein synthesis machinery was also affected by high Ca treatment as increased expression level of ribosomal protein L14 (spot 29) and small subunit ribosomal protein S1 (spot 35) has been detected in *T. wallichiana* var. *mairei* under AR stress. Transcription and translation control the expression of stress responsive genes, which play crucial roles in plants in response to various abiotic stresses (Amme et al., 2006). We detected increased abundance of series transcription and translation-related proteins in *T. wallichiana* var. *mairei* under high Ca-AR treatment, and the results indicated that Ca-regulated transcription and translation mediate *T. wallichiana* var. *mairei* adaptation to AR stress. The complex AR-responsive signaling pathways are associated with stress sensing and activation of defense pathways, which involves in calcium-regulated proteins crosstalk among various transcription factors. Under AR stress, future work on these transcription and translation-related proteins are needed to clarify the specific functions in various Ca conditions.

### Signal Transduction-Related Proteins

Under environmental stresses, woody plants initiate multiple signaling pathways through sensing and transducting several stress signals, further regulate the expression level of many functional proteins, and finally exhibit corresponding physiological response to against the abiotic stresses (Hu et al., 2014a; Liu Y. et al., 2019). Membrane steroid-binding protein 1 (MSBP1) can bind steroids and negatively regulates cell elongation and expansion, and also photomorphogenesis and brassinosteroid signaling (Yang et al., 2005; Song et al., 2009; Shi et al., 2011). In this study, we found that the abundance of membrane steroid-binding protein 1 (spot 37) was significantly increased at low Ca level; however, it can reverse the increased abundance of MSBP1 in *T. wallichiana* var. *mairei* at high Ca level, which provides informative hints on interactions between Ca and membrane steroid-binding protein. This finding suggested the specific relationship between Ca and steroid-binding protein in the BR signaling pathway. Protein phosphatase 2A plays an important role in plant development and growth through a functional link with the target of rapamycin (TOR) signaling pathway (Ahn et al., 2015). The TOR signaling pathway integrates multiple signals transduction, such as energy, nutrients, growth factors, and environmental conditions, to regulate plant cell metabolism and growth (Wullschleger et al., 2006; Xiong et al., 2013). We found that the expression level of 2A phosphatase-associated protein of 46 kDa (spot 38) was significantly upregulated in *T. wallichiana* var. *mairei* with high Ca-AR treatment. On the other hand, it should be noted that the abundance of 2A phosphatase-associated protein of 46 kDa was decreased in *P. massoniana* under high Ca-AR treatment (Hu et al., 2014c), which suggests that 2A phosphatase-associated protein of 46 kDa could play a specific role in woody plants response to AR stress. These results implied the different expression patterns and coping response strategies between AR-sensitive (*P. massoniana*) and AR-resistant (*T. wallichiana* var. *mairei*) tree species under high Ca condition; however, further studies are needed to elucidate the specific function of the signal transduction-related proteins in AR-stressed tree species.

Moreover, Ca plays an important role in plants coping with a series of environmental stresses (Reddy et al., 2011). In the cytoplasm, free cytosolic Ca^2+^ level is a universal second messenger of signal transduction in plants in response to environmental stresses (Kudla et al., 2018). Ca-binding-related proteins regulated modulation of intracellular Ca^2+^ levels (Hu et al., 2014a,^2016^). In this study, expression level of calcium-binding protein KIC-like (spot 40) showed declines in *T. wallichiana* var. *mairei* with high Ca-AR treatment. It has been observed that Ca-binding proteins are regarded as crosstalk key nodes for environmental stress signaling pathways (Hepler, 2005; Liu Y. et al., 2019). These findings suggest that calcium-binding proteins participate in adaptation responses to AR stress in *T. wallichiana* var. *mairei*, which provides informative hints on interactions between Ca-associated signal transduction and AR tolerance. In Figure 7, our result provided additional evidence that the expression abundance of eight Ca-related genes (CaM1, TCH3, CRT3, CDPK1, GDH2, CBL1, CNX1, and RbohA) increased obviously at low Ca level, whereas this expression trend can be reversed at high Ca level under AR stress. Plants respond to environmental stresses through altering gene expression and adaptive responses associated with stress sensing and activation of defense pathways, such as expression of stress adaptive proteins, synthesis of oxidative stress protectors, calcium-regulated proteins, and signal pathway (Hasegawa et al., 2000; Hu et al., 2014a). In this study, these findings indicate that Ca-dependent signal transduction could induce upregulated expression of Ca-related genes and further interact with various biological processes to against AR stress in *T. wallichiana* var. *mairei*.

## Conclusion

**FIGURE 8:**
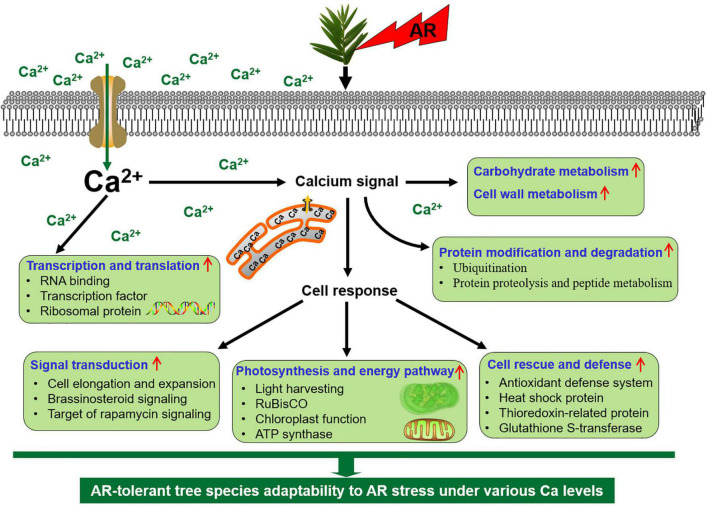
An adaptive strategy of *T. wallichiana* var. *mairei* leaves response to AR stress under different Ca levels.

In this study, physiological and proteomic evidence is carried out to clarify that Ca plays an important role in AR-resistant tree species *T. wallichiana* var. *mairei* response to simulated AR treatment. Our data reveal that low Ca treatment activated the cell rescue- and defense-related proteins and Ca-related gene level to respond to AR stress in *T. wallichiana* var. *mairei*. These proteins might operate in a dynamic network in the response of AR-resistant tree species under AR stress, and these identified proteins might be useful in revealing insights into the different defense mechanisms of AR-resistant tree species to AR stress. In addition, exogenous Ca supply against AR stress by increasing the abundance of the proteins involved in photosynthesis and energy pathway, translational factors, which suggest that AR-tolerant woody plants can equip themselves better to respond to AR stress by provoking related proteins and gene expression. As shown in Figure 8, we proposed a scheme associated with different defense mechanisms of *T. wallichiana* var. *mairei* responding to AR damage at different Ca levels. These results would deepen insights into the adaptation strategies of AR-resistant tree species under AR stress at different Ca levels and furthermore provide theoretical support for the scientific prevention of AR stress.

## Data Availability Statement

The original contributions presented for this study are included in the article/supplementary material. For details, please refer to Supplementary Table S3 and Supplementary Datasheet.

## Author Contributions

H-LZ, W-JH, and G-XS: conceptualization and funding acquisition. W-JH and QW: methodology. T-WL: software and visualization. C-KJ: validation. JW: formal analysis. QW and H-LL: investigation. LC: resources. C-QZ: data curation. W-JH and C-QZ: writing – original draft preparation. H-LZ and G-XS: writing, reviewing, and editing, and project administration. H-LZ: supervision. All authors have read and agreed to the published version of the manuscript.

## Conflict of Interest

The authors declare that the research was conducted in the absence of any commercial or financial relationships that could be construed as a potential conflict of interest.

## Publisher’s Note

All claims expressed in this article are solely those of the authors and do not necessarily represent those of their affiliated organizations, or those of the publisher, the editors and the reviewers. Any product that may be evaluated in this article, or claim that may be made by its manufacturer, is not guaranteed or endorsed by the publisher.

## Footnotes

1
http://www.ncbi.nlm.nih.gov

2
http://www.uniprot.org
