# Supplementary material for: Physiological, Proteomic Analysis, and Calcium-Related Gene Expression Reveal Taxus wallichiana var. mairei Adaptability to Acid Rain Stress Under Various Calcium Levels
Source: Front Plant Sci. 2022 Mar 21;13:845107. doi: 10.3389/fpls.2022.845107 (PMC8978443; doi:10.3389/fpls.2022.845107)
Supplement: Supplementary file 1 [file Table_1.DOC]

*Table S1. Primer sequences used for cloning the fragments of Ca-related genes in Taxus wallichiana var. mairei.*

| Ca-related gene | Degenerate primer sequences |
| --- | --- |
| *CaM1* | F:5'AGGARGCYTTYAGYYTVTTYGAYAARGA3' |
|  | R:5'ATCTCATCMACYTCYTCRTCVGTDAGC3' |
| *GDH2* | F:5'ATGAGGTNAATGCHTTRGCACAACT3' |
|  | R:5'ATGTTCTGVACCCACTCAAARTAGC3' |
| *TCH3* | F:5'TTGGNACNGTGATGAGGTCNYTGGG3' |
|  | R:5'ATCATYTCATCNACTTCTTCATCA3' |
| *CBL1* | F:5'TGAAGCAYTDTTYGAACTDTACAAG3' |
|  | R:5'ACCTCTTBNCGYTCAATRAANCCHG3' |
| *CRT3* | F:5'ACCTCTTBNCGYTCAATRAANCCHG6' |
|  | R:5'CCCTCWGGYTTCTTRTCNTCWGGRTC3' |
| *CNX1* | F:5'GGTKCATTTCATYYTNAAGCACAAG3' |
|  | R:5'CACACTTBGGGTTDTCAATCTTTGG' |
| *RbohA* | F:5'CTTTYGATGAYAAYCTYAATTTYCA' |
|  | R:5'ARTTATCTTCWGGDGCYGAAGTAAT' |
| *CDPK1* | F:5'AGYTDTGTGCHGGNGGTGARCTNTT3' |
|  | R:5'TCDGCCCARAANGGAGGHACNCCAC3' |
| *18S* | F:5'GGCTYGTCCCTTCTGYCGGCGATRC3' |
|  | R:5'ATTGCCTCAAACTTCCKTGGCCTAA3' |
