# Supplementary material for: Physiological, Proteomic Analysis, and Calcium-Related Gene Expression Reveal Taxus wallichiana var. mairei Adaptability to Acid Rain Stress Under Various Calcium Levels
Source: Front Plant Sci. 2022 Mar 21;13:845107. doi: 10.3389/fpls.2022.845107 (PMC8978443; doi:10.3389/fpls.2022.845107)
Supplement: Supplementary file 2 [file Table_2.DOC]

Table S2. Primer sequences used for qRT-PCR of Ca-related genes in *Taxus wallichiana* var. *mairei*.

| Ca-related gene | Primer sequences |
| --- | --- |
| *CaM1* | F:5'AAGGATACGGACTCAGAGGAAGAAT3' |
|  | R:5'GCTTCACGAATCATCTCATCCACCT3' |
| *GDH2* | F:5'TCAGAAGATACACGACTTAGTTGGA3' |
|  | R:5'GACAGGTTTTCCCGTTACGATTGCG3' |
| *TCH3* | F:5'TGAAGGATACGGACTCAGAGGAAG3' |
|  | R:5'TTCTCCCCCAGGTTAGTCATCACAT3' |
| *CBL1* | F:5'AATCTTTTTGCTGATAGGGTCTTCG3' |
|  | R:5'CCGTTCAATAAATCCCGTCTGTC3' |
| *CRT3* | F:5'CAAAGGGAAGAACCAACCTATCA3' |
|  | R:5'ACTCCCAGATTCCTTTTCAGTGTTG3' |
| *CNX1* | F:5'CCTCCCTCTGTTCCATCTGATAAGC3' |
|  | R:5'AGTTTTTGGTGGGATTAGAGCAGGC3' |
| *RbohA* | F:5'CTCAGCGTCCTCTCTCCCCCGTAT3' |
|  | R:5'TCTGGTATTCTCATCATCTTTTCG3' |
| *CDPK1* | F:5'CCAGGTGTTCAGTGATGTAGTTG3' |
|  | R:5'TGGAGGTACGCCACTCAGTAAGATG3' |
| *GAPDH* | F:5'CGGAGACAGTCGATCAAGC3' |
|  | R:5'CCCTACCTCAACCCAATAA3' |
