# Supplementary material for: Physiological, Proteomic Analysis, and Calcium-Related Gene Expression Reveal Taxus wallichiana var. mairei Adaptability to Acid Rain Stress Under Various Calcium Levels
Source: Front Plant Sci. 2022 Mar 21;13:845107. doi: 10.3389/fpls.2022.845107 (PMC8978443; doi:10.3389/fpls.2022.845107)
Supplement: Supplementary file 3 [file Table_3.docx]

Table S3. Lists of all identified peptides in differentially expressed proteins.

| **Spot^a^** | **NCBI  accession^b^** | **Protein identity^c^** | **Pep. Count^d^** | **Peptide sequence** |
| --- | --- | --- | --- | --- |
| 1 | gi\|2500930 | cell wall beta-fructosidase 1 | 6 | SPKNPLMEPTIANK |
|  |  |  |  | NPLMEPTIANK |
|  |  |  |  | HPLHSAEGTGMWECPDFYPVLDK |
|  |  |  |  | HPLHSAEGTGMWECPDFYPVLDKNLLR |
|  |  |  |  | GAGVKGGVGPFGLLVFASQGLK |
|  |  |  |  | VYPTLAIHDK |
| 2 | gi\|158705664 | UDP-glucose pyrophosphorylase | 6 | LDQISENEKAGFTSLVSR |
|  |  |  |  | IQTPTDEVVVPYDTLAPAPEDLDATK |
|  |  |  |  | LDTLLSQGKEYVFVANSDNLGAIVDIK |
|  |  |  |  | VQLLEIAQVPDEHVNEFKSIEK |
|  |  |  |  | IFNTNNLWVNLK |
|  |  |  |  | ATSDLLLVQSDLYTLVDGFVISNPAR |
| 3 | gi\|269980525 | IAA-amino acid hydrolase | 6 | LSHLTRELLESAR |
|  |  |  |  | ADMDALPIQEMVEWEHK |
|  |  |  |  | ADMDALPIQEMVEWEHKSK |
|  |  |  |  | NNGKMHACGHDAHVTMLLGAAK |
|  |  |  |  | MRPYPSTVNDEAMYKHAK |
|  |  |  |  | HAIDTQ |
| 4 | gi\|223635315 | S-adenosylmethionine synthase | 7 | VACETCTKTNMVMVFGEITTK |
|  |  |  |  | TNMVMVFGEITTK |
|  |  |  |  | KNATCPWLRPDGK |
|  |  |  |  | EHVIKPVIPEQYLDENTIFHLNPSGR |
|  |  |  |  | VDRSGAYVAR |
|  |  |  |  | SIVASGIAR |
|  |  |  |  | EGADFTWEVVKPLK |
| 5 | gi\|77556698 | phytoene synthase | 5 | MASSSSAAALWTAAPHPHGSCIR |
|  |  |  |  | QAALVEEATHR |
|  |  |  |  | RGAGAPR |
|  |  |  |  | RLEDLFEGRPYDMYDAALSDTVSK |
|  |  |  |  | VTDKWR |
| 6 | gi\|385178691 | probable aspartyl aminopeptidase | 6 | YVAGNGFYVVGAHTDSPCIKLKPVSK |
|  |  |  |  | LVRIEEPIMR |
|  |  |  |  | VNTQSHLLPVLATSVK |
|  |  |  |  | MHGGLVIKHNANQR |
|  |  |  |  | TVDVGAPQLSMHSIR |
|  |  |  |  | TVDVGAPQLSMHSIREMCAVDDVK |
| 7 | gi\|138277483 | ATP synthase beta subunit | 18 | IAQIIGPVLDVSFPPGNMPK |
|  |  |  |  | GMIVIDTGAPLSVPVGETTLGR |
|  |  |  |  | GMIVIDTGAPLSVPVGETTLGR + Oxidation (M) |
|  |  |  |  | IFNVLGEPVDDLGPVNALTTSPIHR |
|  |  |  |  | FSIFETGIK |
|  |  |  |  | VVDLLAPYR |
|  |  |  |  | AHGGVSVFGGVGER |
|  |  |  |  | VALVYGQMNEPPGAR |
|  |  |  |  | VALVYGQMNEPPGAR + Oxidation (M) |
|  |  |  |  | VGLTALTMAEYFR |
|  |  |  |  | VGLTALTMAEYFR + Oxidation (M) |
|  |  |  |  | DVNKQDVLLFIDNIFR |
|  |  |  |  | QDVLLFIDNIFR |
|  |  |  |  | FVQAGSEVSALLGR |
|  |  |  |  | MPSAVGYQPTLSTEMGSLQER |
|  |  |  |  | YKELQDIIAILGLDELSEEDR |
|  |  |  |  | ELQDIIAILGLDELSEEDR |
|  |  |  |  | ELQDIIAILGLDELSEEDRLTVAR |
| 8 | gi\|138277483 | ATP synthase beta subunit | 18 | IAQIIGPVLDVSFPPGNMPK |
|  |  |  |  | GMIVIDTGAPLSVPVGETTLGR |
|  |  |  |  | GMIVIDTGAPLSVPVGETTLGR + Oxidation (M) |
|  |  |  |  | IFNVLGEPVDDLGPVNALTTSPIHR |
|  |  |  |  | FSIFETGIK |
|  |  |  |  | VVDLLAPYR |
|  |  |  |  | AHGGVSVFGGVGER |
|  |  |  |  | VALVYGQMNEPPGAR |
|  |  |  |  | VGLTALTMAEYFR |
|  |  |  |  | VGLTALTMAEYFR + Oxidation (M) |
|  |  |  |  | DVNKQDVLLFIDNIFR |
|  |  |  |  | QDVLLFIDNIFR |
|  |  |  |  | FVQAGSEVSALLGR |
|  |  |  |  | MPSAVGYQPTLSTEMGSLQER |
|  |  |  |  | MPSAVGYQPTLSTEMGSLQER + Oxidation (M) |
|  |  |  |  | YKELQDIIAILGLDELSEEDR |
|  |  |  |  | ELQDIIAILGLDELSEEDR |
|  |  |  |  | ELQDIIAILGLDELSEEDRLTVAR |
| 9 | gi\|4388533 | F1-ATP synthase beta subunit | 16 | LVLEVAQHLGENMVR |
|  |  |  |  | LVLEVAQHLGENMVR + Oxidation (M) |
|  |  |  |  | VLNTGSPITVPVGRATLGR |
|  |  |  |  | VVDLLAPYQR |
|  |  |  |  | TVLIMELINNVAK |
|  |  |  |  | AHGGFSVFAGVGER |
|  |  |  |  | LGDKQSESK |
|  |  |  |  | CALVYGQMNEPPGAR |
|  |  |  |  | CALVYGQMNEPPGAR + Oxidation (M) |
|  |  |  |  | CALVYGQMNEPPGARAR |
|  |  |  |  | VGLTGLTVAEHFR |
|  |  |  |  | DAEGQDVLLFIDNIFR |
|  |  |  |  | FTQANSEVSALLGR |
|  |  |  |  | IPSAVGYQPTLATDLGGLQER |
|  |  |  |  | QISELGIYPAVDPLDSTSR |
|  |  |  |  | NLQDIIAILGMDELSEDDKLTVAR |
| 10 | gi\|226493589 | ATP synthase beta chain | 12 | AASPAAPRPRGAPHRPSPAGYLFNR |
|  |  |  |  | LVLEVAQHLGENMVR |
|  |  |  |  | VVDLLAPYQR |
|  |  |  |  | AHGGFSVFAGVGER |
|  |  |  |  | CALVYGQMNEPPGAR |
|  |  |  |  | CALVYGQMNEPPGARAR |
|  |  |  |  | VGLTGLTVAEHFR |
|  |  |  |  | DAEGQDVLLFIDNIFR |
|  |  |  |  | FTQANSEVSALLGR |
|  |  |  |  | IPSAVGYQPTLATDLGGLQER |
|  |  |  |  | QISELGIYPAVDPLDSTSR |
|  |  |  |  | NLQDIIAILGMDELSEDDKLTVAR |
| 11 | gi\|7592732 | plasma membrane H^+^-ATPase | 6 | TGTLTLNKLTVDK |
|  |  |  |  | GIDANTVVLMAAR |
|  |  |  |  | TALTYIDGQGQMHR |
|  |  |  |  | GAPEQILNMAHNK |
|  |  |  |  | VHAVIDKFAER |
|  |  |  |  | SLAVAYQEVTEKR |
| 12 | gi\|372486191 | NADH-plastoquinone oxidoreductase subunit 7 | 6 | MTVPATR |
|  |  |  |  | ELLYDLFEAATGMRMMHNYFR |
|  |  |  |  | MMHNYFRIGGVAADLPYGWIDK |
|  |  |  |  | VEGVGIIGVEEAINWGLSGPMLR |
|  |  |  |  | FDWEVQWQKEGDSLAR |
|  |  |  |  | EGDSLAR |
| 13 | gi\|311893429 | ATP-dependent zinc metalloprotease ThFtsH8 | 10 | MAASSACLIGSGLSVHTTK |
|  |  |  |  | QLGLSSTFASVDR |
|  |  |  |  | VTVVKASLDVK |
|  |  |  |  | SSGGMGGPGGPGFPLQLGQSK |
|  |  |  |  | AIAGEAGVPFFSISGSEFVEMFVGVGASR |
|  |  |  |  | AKENAPCIVFVDEIDAVGR |
|  |  |  |  | ENAPCIVFVDEIDAVGR |
|  |  |  |  | KFENGVSLEVIAMR |
|  |  |  |  | TPGFSGADLANLLNEAAILAGR |
|  |  |  |  | QQLFAR |
| 14 | gi\|356517518 | ATP-dependent zinc metalloprotease FTSH | 14 | MAFATSALVCSNLLGRK |
|  |  |  |  | YSDFLNAVK |
|  |  |  |  | RAQGGPGGPGGMGGPMDFGR |
|  |  |  |  | FQEVPETGVSFADVAGADQAK |
|  |  |  |  | LELQEVVDFLK |
|  |  |  |  | IPKGCLLVGPPGTGK |
|  |  |  |  | APCIVFIDEIDAVGR |
|  |  |  |  | TPGFTGADLQNLMNEAAILAAR |
|  |  |  |  | EISKDEISDALER |
|  |  |  |  | LVAYHEAGHALVGALMPEYDPVAK |
|  |  |  |  | GQAGGLTFFAPSEER |
|  |  |  |  | SYLENQMAVALGGR |
|  |  |  |  | SYLENQMAVALGGR + Oxidation (M) |
|  |  |  |  | ETVDGEEFMSLFIDGK |
| 15 | gi\|3293555 | chlorophyll a/b binding protein | 6 | MAASSMALSSPTLAGKPVK |
|  |  |  |  | ELEVIHSR |
|  |  |  |  | WAMLGALGCVFPELLSR |
|  |  |  |  | WAMLGALGCVFPELLSR + Oxidation (M) |
|  |  |  |  | FGEAVWFK |
|  |  |  |  | GPLENLADHLADPVNNNAWAYATNFVPGK |
| 16 | gi\|91177512 | ribulose-1,5-bisphosphate carboxylase/oxygenase large subunit | 16 | DTDILAAFR |
|  |  |  |  | IPISYIKTFQGPPHGIQVER |
|  |  |  |  | YGRPLLGCTIKPK |
|  |  |  |  | AVYECLR |
|  |  |  |  | GGLDFTKDDENVNSQPFMR |
|  |  |  |  | DDENVNSQPFMR |
|  |  |  |  | FCFCAEALYK |
|  |  |  |  | ELGVPIVMHDYLTGGFTANTSLAHYCR |
|  |  |  |  | DNGLLLHIHR |
|  |  |  |  | QKNHGMHFR |
|  |  |  |  | NHGMHFR |
|  |  |  |  | MSGGDHIHAGTVVGK |
|  |  |  |  | DITLGFVDLLR |
|  |  |  |  | DITLGFVDLLRDDFIEK |
|  |  |  |  | VALEACVKAR |
|  |  |  |  | WSPELAAACEVWK |
| 17 | gi\|224114357 | light-harvesting complex II protein Lhcb1 | 7 | MAAATMALSSPAFAGKAVK |
|  |  |  |  | AAATMALSSPAFAGKAVK |
|  |  |  |  | KTTKPVSSGSPWYGPDR |
|  |  |  |  | WAMLGALGCVFPELLSR |
|  |  |  |  | WAMLGALGCVFPELLSR + Oxidation (M) |
|  |  |  |  | FGEAVWFK |
|  |  |  |  | LAMFSMFGFFVQAIVTGK |
| 18 | gi\|3913651 | ferredoxin-NADP reductase | 6 | MATAVSAAVSLPSSKSTSFSSR |
|  |  |  |  | INFNKVPLYYR |
|  |  |  |  | EMLMPKDPNATVIMLATGTGIAPFR |
|  |  |  |  | HEDYKFNGTAWLFLGVPTSSSLLYK |
|  |  |  |  | MYIQTRMAQYAEELWTLLQK |
|  |  |  |  | GMEQGIDEIMSALAERDGIVWADYK |
| 19 | gi\|255570990 | heat shock protein | 8 | VVGIDLGTTNSAVAAMEGGKPTIVTNAEGQR |
|  |  |  |  | QFAAEEISAQVLR |
|  |  |  |  | IAGLEVLR |
|  |  |  |  | QALQRLTETAEK |
|  |  |  |  | MELSSLTQTNISLPFITATADGPK |
|  |  |  |  | AKFEELCSDLLDR |
|  |  |  |  | TPVENSLR |
|  |  |  |  | ELKDAIEGGSTQGMK |
| 20 | gi\|392465167 | heat shock protein 70 | 17 | GEGPAIGIDLGTTYSCVGVWQHDR |
|  |  |  |  | VEIIANDQGNR |
|  |  |  |  | TTPSYVGFTDTER |
|  |  |  |  | QFAAEEISSMVLIK |
|  |  |  |  | NAVVTVPAYFNDSQR |
|  |  |  |  | DAGVIAGLNVLR |
|  |  |  |  | IINEPTAAAIAYGLDKK |
|  |  |  |  | ATAGDTHLGGEDFDNR |
|  |  |  |  | MVNHFVQEFKR |
|  |  |  |  | TLSSTAQTTIEIDSLYEGIDFYSTITR |
|  |  |  |  | ARFEELNMDLFR |
|  |  |  |  | FEELNMDLFR |
|  |  |  |  | FEELNMDLFR + Oxidation (M) |
|  |  |  |  | CMEPVEKCLR |
|  |  |  |  | STIHDVVLVGGSTR |
|  |  |  |  | SINPDEAVAYGAAVQAAILSGEGNEK |
|  |  |  |  | NALENYAYNMR |
| 21 | gi\|357493781 | thioredoxin-related protein | 5 | GTAPQNTFPSR |
|  |  |  |  | SGEFNQMPDLFDAIHAFVGHR |
|  |  |  |  | GSDNRFGLMDAVDNFMR |
|  |  |  |  | GDFGDYFMGSGLEELIEQLTMNDR |
|  |  |  |  | VELPPQGQMSSRGSSR |
| 22 | gi\|110808557 | trypsin inhibitor AeTI | 3 | AKELLDSDGIILR |
|  |  |  |  | ELLDSDGIILR |
|  |  |  |  | ELLDSDGIILRNGANYYI |
| 23 | gi\|153865891 | alcohol dehydrogenase 1 | 5 | GSTVAIFGLGAVGLAAAEGAR |
|  |  |  |  | IIGVDLNPSRFEEAR |
|  |  |  |  | MFGCTEFVNPK |
|  |  |  |  | DAEFKTHPMNFLNER |
|  |  |  |  | THPMNFLNERTLK |
| 24 | gi\|327342604 | glutathione S-transferase | 6 | SDVKLIGAWPSPFVMRPR |
|  |  |  |  | SEGYEFLEETLGSK |
|  |  |  |  | FWAAYLDEKWFPTMR |
|  |  |  |  | SIATAGEEAARK |
|  |  |  |  | TNGVKLIDETK |
|  |  |  |  | LIDETKTPSLFK |
| 25 | gi\|335346406 | abscisic acid 8-hydroxylase | 7 | MEFIMVMLFTLATIFFLK |
|  |  |  |  | MEFIMVMLFTLATIFFLKK |
|  |  |  |  | YGSIFK |
|  |  |  |  | THILGCPCVMISSPEAAKVVLVTK |
|  |  |  |  | ERMLGK |
|  |  |  |  | LVLRAFMPDALK |
|  |  |  |  | GKEGQVLCWEHTK |
| 26 | gi\|302815799 | 2-oxoglutarate-iron(II)-dependent oxygenase | 6 | VLVDGGMDIVPDIYIQPEHQR |
|  |  |  |  | ELSNSGNRPKAIAAIGQACQK |
|  |  |  |  | MKEVAYEFFELPVEEK |
|  |  |  |  | HSCNPLSEENVSSWPANPPSYR |
|  |  |  |  | QATHSYSEAVGSLCKSLLR |
|  |  |  |  | RFYAK |
| 27 | gi\|14579025 | maturase K | 10 | LVGHNKSFYSELVLGGLTAVLEVPFSIR |
|  |  |  |  | RWIQDAPFLHSLR |
|  |  |  |  | LSLFLWNYYAYECESLLVPLWK |
|  |  |  |  | SLPYESFIERTPFYR |
|  |  |  |  | DPSIHYVKYR |
|  |  |  |  | MKTAVVR |
|  |  |  |  | NIFHYHSGSSKK |
|  |  |  |  | SFPKER |
|  |  |  |  | IWHSDIIQR |
|  |  |  |  | IWHSDIIQRNPLVNSWWK |
| 28 | gi\|56744289 | putative transposon MuDR mudrA-like protein | 9 | IGDFYMLK |
|  |  |  |  | LHQIQGLVQKDYGLYVSK |
|  |  |  |  | DYGLYVSK |
|  |  |  |  | NGYTTGTAR |
|  |  |  |  | SGGATILAASTGVTAGGSDGATTK |
|  |  |  |  | RPATTSATSRGATIGGR |
|  |  |  |  | GGANPEYKRPR |
|  |  |  |  | QLQEQSYK |
|  |  |  |  | QLQEQSYKR |
| 29 | gi\|67968326 | ribosomal protein L14 | 5 | TIRPQTYSNAADNSGAR |
|  |  |  |  | TIRPQTYSNAADNSGARK |
|  |  |  |  | SMCARAPGASNR |
|  |  |  |  | DNGAAVRSDDNAAVAIDR |
|  |  |  |  | ESRECHFAK |
| 30 | gi\|62733886 | retrotransposon protein | 7 | MAPAQPAPPAQAK |
|  |  |  |  | QGASLNLVEAKSSVTAEEK |
|  |  |  |  | RYIRPSTSPWGAPIIFVEK |
|  |  |  |  | EEDIPKSGEEHEQHLR |
|  |  |  |  | CDFWLSEVK |
|  |  |  |  | LVSAQEELLQR |
|  |  |  |  | MYQDLKEK |
| 31 | gi\|379054892 | initiation factor 4A-3-like protein | 10 | GIYAYGFEKPSAIQQR |
|  |  |  |  | ALGDYLGVKVHACVGGTSVR |
|  |  |  |  | VFDMLR |
|  |  |  |  | MFVLDEADEMLSR |
|  |  |  |  | GFKDQIYDIFQLLPAK |
|  |  |  |  | IQVGVFSATMPPEALEITR |
|  |  |  |  | LDTLCDLYETLAITQSVIFVNTR |
|  |  |  |  | VLITTDLLAR |
|  |  |  |  | GIDVQQVSLVINYDLPTQPENYLHR |
|  |  |  |  | KGVAINFVTR |
| 32 | gi\|359494561 | transcription factor JUNGBRUNNEN 1 | 6 | MDATKDDDQVVLPGFR |
|  |  |  |  | TDWMMHEFR |
|  |  |  |  | TDWMMHEFRLPANQK |
|  |  |  |  | AADQEAEIWTLCR |
|  |  |  |  | ESSARHNPVNAR |
|  |  |  |  | IQLHVGSEPESCSTVLSEEAR |
| 33 | gi\|159480324 | mitochondrial transcription termination factor | 6 | MSAMLVGRQGLGVACSSR |
|  |  |  |  | SAMLVGR |
|  |  |  |  | SAMLVGRQGLGVACSSR |
|  |  |  |  | GVPDSGIPDLVLK |
|  |  |  |  | IFEYKLSADGSELVK |
|  |  |  |  | EGASFMEAPVSPVGPVRAP |
| 34 | gi\|77551510 | transposon protein | 10 | MSQQGEPMSTTTIGTTSSRNPR |
|  |  |  |  | DHIDLVPQEEKEEAWR |
|  |  |  |  | QAIRNMGNCWK |
|  |  |  |  | YAFITQPVWDEFHAAK |
|  |  |  |  | QASQEVCPQK |
|  |  |  |  | HSIEINITPR |
|  |  |  |  | LAPSSRPPPTRPHQTVVSLPSAVEQR |
|  |  |  |  | AKSSPPK |
|  |  |  |  | APVAPPKPLAKFILGMPLVGDDALFK |
|  |  |  |  | ANIVAYIKDCLFAR |
| 35 | gi\|334183835 | small subunit ribosomal protein S1 | 8 | MQTLLCQPCKSLPILTASSSSSLIR |
|  |  |  |  | MGIVKDDDEGVEIAEFAR |
|  |  |  |  | DDDEGVEIAEFAR |
|  |  |  |  | IAWHRVR |
|  |  |  |  | FLVQITRLNEDK |
|  |  |  |  | SGLLHISNITR |
|  |  |  |  | VFTEAEEMAK |
|  |  |  |  | DEEIYANWEWFK |
| 36 | gi\|357469355 | F-box/kelch-repeat protein | 7 | SKNEEEDMQNNATFPVHSIDNGNGK |
|  |  |  |  | YNFLTNMWSVGK |
|  |  |  |  | CLFGSASLGEIAILAGGCDPR |
|  |  |  |  | EIPNMFPMPTGVLEAPPSYGPPPLIAVVK |
|  |  |  |  | FPEQATSMKGWGLAFR |
|  |  |  |  | KIGSFVYSCTVMGCLMMFSVA |
|  |  |  |  | IGSFVYSCTVMGCLMMFSVA |
| 37 | gi\|195627742 | membrane steroid-binding protein 1 | 4 | AYDGTDPSKPIYVSVRGK |
|  |  |  |  | VYDVTSGRGFYGPGGAYAVFAGR |
|  |  |  |  | MSKDEADVSGDLSGLTDK |
|  |  |  |  | ELGVLADWETK |
| 38 | gi\|356516069 | 2A phosphatase-associated protein of 46 kDa | 11 | GCEALHR |
|  |  |  |  | GCEALHRCEDMVNNLGLFSPNETK |
|  |  |  |  | EFISFCEAMELVPK |
|  |  |  |  | KEEEMLSAVK |
|  |  |  |  | EEEMLSAVK |
|  |  |  |  | EEEMLSAVK + Oxidation (M) |
|  |  |  |  | MAAQVFQPSHR |
|  |  |  |  | MAAQVFQPSHR + Oxidation (M) |
|  |  |  |  | MPTMSIEEAGLKEMEIMNK |
|  |  |  |  | EMEIMNKWQER |
|  |  |  |  | LMEEANSSWHNDRK |
| 39 | gi\|307939386 | lectin | 4 | FIAATNVLKAR |
|  |  |  |  | VTYSNSHSYNHSDVVPLK |
|  |  |  |  | VTYSNSHSYNHSDVVPLKNIVPEWVR |
|  |  |  |  | NIVPEWVR |
| 40 | gi\|356573251 | calcium-binding protein KIC-like | 7 | EITVEVEEFEDLLPVMAKK |
|  |  |  |  | NSTLLGMDGMSKEEAETMVR + Oxidation (M) |
|  |  |  |  | NSTLLGMDGMSKEEAETMVR + 2 Oxidation (M) |
|  |  |  |  |  |
|  |  |  |  |  |
|  |  |  |  |  |
|  |  |  |  | EEAETMVRQGDLDGDGK |
|  |  |  |  | QGDLDGDGK |
|  |  |  |  | QGDLDGDGKLNETEFCILMVR |
|  |  |  |  | LNETEFCILMVR |
| 41 | gi\|116782579 | unknown | 4 | CEAEENQGAGAGDK |
|  |  |  |  | AATYGSIGMSGRAMGK |
|  |  |  |  | LCEAEENQGAGAGDK |
|  |  |  |  | MCEAEGLWEVMLK |
| 42 | gi\|356566253 | uncharacterized protein LOC100799858 | 6 | IDIPSLASMKEQMTTIMEAMMDMR |
|  |  |  |  | EQMTTIMEAMMDMR |
|  |  |  |  | GTACPKNHLR |
|  |  |  |  | NHLRMYYK |
|  |  |  |  | MYYKR |
|  |  |  |  | DLMDAFSR |
| 43 | gi\|296087931 | unnamed protein product | 11 | MTHSQK |
|  |  |  |  | DVAYHAEDLLDEIATEALR |
|  |  |  |  | AWNWEKVSTWVK |
|  |  |  |  | IVVTSRSETAAK |
|  |  |  |  | SETAAKIMR |
|  |  |  |  | DYEFDKEK |
|  |  |  |  | LQKISDK |
|  |  |  |  | AVVFETFESVK |
|  |  |  |  | LPESICCLCNLQTMMLSKCR |
|  |  |  |  | YLDISGSNSLKEMPNDIDQLK |
|  |  |  |  | LEISKMENVVGVEDALQAHMK |
| 44 | gi\|20198271 | hypothetical protein | 5 | GLWDILPVDAENLAK |
|  |  |  |  | GLWDILPVDAENLAKK |
|  |  |  |  | VDNTVLWHQRLGHMSQK |
|  |  |  |  | LGHMSQKNMDLLVK |
|  |  |  |  | LDYVHSDLWGAPTVPLSL |

^a^Spot. is the unique differentially expressed protein spot number.

^b^Database accession numbers according to NCBInr.

^c^The name of the proteins identified by MALDI-TOF/TOF MS.

^d^Number of the matched peptides.
